# Visible light assisted photocatalytic degradation of crystal violet dye and electrochemical detection of ascorbic acid using a BiVO_4_/FeVO_4_ heterojunction composite†

**DOI:** 10.1039/c8ra03890b

**Published:** 2018-06-27

**Authors:** Muhammad Munir Sajid, Sadaf Bashir Khan, Naveed Akthar Shad, Nasir Amin, Zhengjun Zhang

**Affiliations:** Department of Physics, Government College University Allama Iqbal Road Faisalabad 38000 Pakistan; National Institute for Biotechnology and Genetic Engineering (NIBGE) P. O. Box. 577, Jhang Road Faisalabad Pakistan; The State Key Laboratory for New Ceramics & Fine Processing, School of Materials Science & Engineering, Tsinghua University Beijing China 100084; Advanced Key Laboratory for New Ceramics, School of Materials Science & Engineering, Tsinghua University Beijing China 100084 zjzhang@tsinghua.edu.cn

## Abstract

A BiVO_4_/FeVO_4_ nanocomposite photocatalyst was successfully synthesized *via* a hydrothermal method. The prepared heterojunction photocatalyst was characterized physically and chemically using XRD, SEM, EDX, XPS, BET, FT-IR, Raman, UV-vis DRS, EPR and photoluminescence techniques. BiVO_4_/FeVO_4_ was explored for its photocatalytic activity by the decomposition of crystal violet (CV) organic dye under visible radiation. This experiment showed that BiVO_4_/FeVO_4_ at a ratio of 2 : 1 completely degrades CV within 60 min. In addition, BiVO_4_/FeVO_4_ was investigated for the electrochemical detection of the useful analyte ascorbic acid using electrochemical impedance spectroscopy (EIS) and cyclic voltammetry techniques. This work reveals the potential of the BiVO_4_/FeVO_4_ nanocomposite for applications in environmental disciplines as well as in biosensing.

## Abstract 2

A highly pure BiVO_4_/FeVO_4_ heterojunction nanocomposite photocatalyst was synthesized by a facile hydrothermal method. The simple and economical manufacturing process allows easier industrial scale-up and the results contribute to the design of more effective photocatalysts.

## Graphical abstract

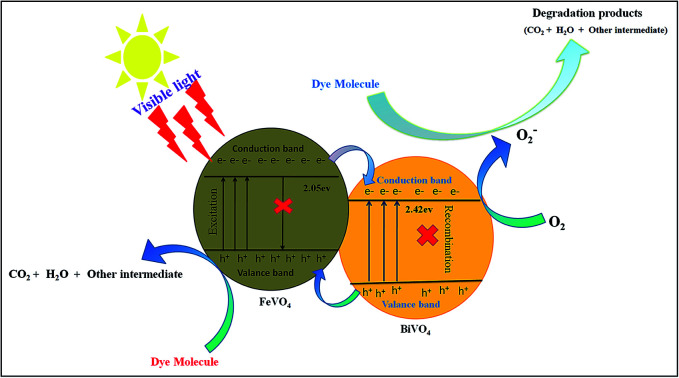

## Introduction

The large-scale disposal of dyes from fabrics, plastics, cosmetics, paper, leather, food, municipal waste and other industries into the water poses a significant threat to the environment. Approximately 1–20% of the dye produced globally is lost during the dyeing process and is expelled in waste water.^1–5^ These dyes are major causes of water pollution, so it is urgent that we develop either facile methods or eco-friendly techniques to treat polluted water and make it usable for human beings and other living organisms. A lot of work has been conducted in this area, involving approaches including filtration, electrochemical methods, precipitation, coagulation and adsorption, which are all common techniques for water remediation.^6^ Among these processes, heterogeneous photocatalysis is an advanced oxidant process (AOP) that has emerged in recent years. AOPs are used extensively as a facile and cost-effective way to mineralize pollutant water without generating secondary harmful pollutants, by using light in the presence of a catalyst.^7–9^ The effectiveness of an AOP is proportional to its ability to generate hydroxyl radicals. Photocatalysis is based on the principle in which the absorption of sunlight energy produces electrons and holes to activate oxidation–reduction responses on the surface of a semiconductor to degrade particular compounds.

Metallic oxide (MO) semiconductors are generally considered as photocatalysts, but because of their wide band gaps, the light absorption ability of binary MOs is limited, affecting their photocatalytic efficiency. Therefore, it is necessary to fabricate MOs with smaller band gaps for effective employment of solar energy. To this end, ternary metal oxides (TMOs), which have valence bands made from orbitals of more than one element, come forward. TMOs have narrow band gaps with high visible light illumination absorption ability. Among these TMOs, metal vanadate is of great importance in several areas including photocatalysis, catalysis and batteries.^10–13^ One of the various metal vanadates that a lot of effort has been devoted to synthesizing and characterizing is bismuth vanadate (BiVO_4_), owing to its good optical, conductivity and ferroelasticity properties. It is used in yellow pigments, O_2_ evaluation, degradation of pollutants, electrodes for batteries and gas sensors.^14–17^

Bismuth vanadate was first reported as a photocatalyst in 1998 for water splitting by Kudo and Ueda, with the monoclinic-scheelite (m-BiVO_4_) phase showing the highest efficiency. In the next year, Kudo's group described the increased visible radiation photocatalytic attributes of BiVO_4_ for O_2_ evolution in an aqueous silver nitrate solvent. In another exciting investigation reported by Kudo *et al.*, monoclinic and tetragonal BiVO_4_, which both have a scheelite structure, and the same composition, components and energy structure, showed extensively divergent photocatalytic efficiencies for O_2_ evolution in an AgNO_3_ mixture under ultraviolet and visible light, owing to the distortion of the Bi–O polyhedron due to the 6S_2_ lone pairs of Bi^3+^.^18–20^

Fabricating heterojunction photocatalysts based on various semiconductor materials represents an economical process to aid the separation of photo-excited charges (electron–hole pairs (ehps)) and increase the photocatalytic properties.^21^ Heterojunction photocatalysts are manufactured in several different ways, including sol–gel synthesis, ball milling,^22^ hydrothermal synthesis^23^ and co-precipitation,^24^ which lead to a heterojunction photocatalyst with an improved photocatalytic response as compared to that of its single ingredients. From a literature survey, it was found that heterojunction photocatalysts such as InVO_4_/BiVO_4_, CuO/BiVO_4_, C_3_N_4_/BiVO_4_, BiVO_4_/Bi_2_S_3_ and CaFe_2_O_4_/Ag_3_VO_4_ exhibit higher photocatalytic efficiency compared to single semiconductors.^25–29^ When a large band gap semiconductor is combined with a short band gap semiconductor, the electron–hole separation is prolonged and a superior photocatalytic response is achieved.^22^

To the best of our knowledge, there are few reports in the literature regarding the use of BiVO_4_/FeVO_4_ nanostructured heterojunction composites for the photocatalytic degradation of crystal violet dye, as well as the electrochemical detection of ascorbic acid. In this study, a BiVO_4_/FeVO_4_ heterojunction composite was prepared *via* a hydrothermal method. Both BiVO_4_ and FeVO_4_ are earth-abundant minerals with narrow band gaps and show innate visible light absorption at around 600 nm at about 2.05 eV. Therefore, combining BiVO_4_ and FeVO_4_ can increase the extent of electron and hole separation and enhance the photocatalytic process. The as-synthesized BiVO_4_/FeVO_4_ nanocomposite was investigated using XRD, SEM, EDS, XPS, BET, UV-vis and photoluminescence techniques for physical and chemical behavior studies. The photocatalytic response of the as-synthesized nanocomposite was evaluated by investigating the degradation of crystal violet (CV) dye under visible light. The electrochemical properties of the BiVO_4_/FeVO_4_ nanocomposite were also investigated for the detection of the important analyte ascorbic acid. This work exposes the potential of the BiVO_4_/FeVO_4_ nanocomposite for applications in environmental science as well as biosensor domains.

## Experimental methods

**Fig. 1:**
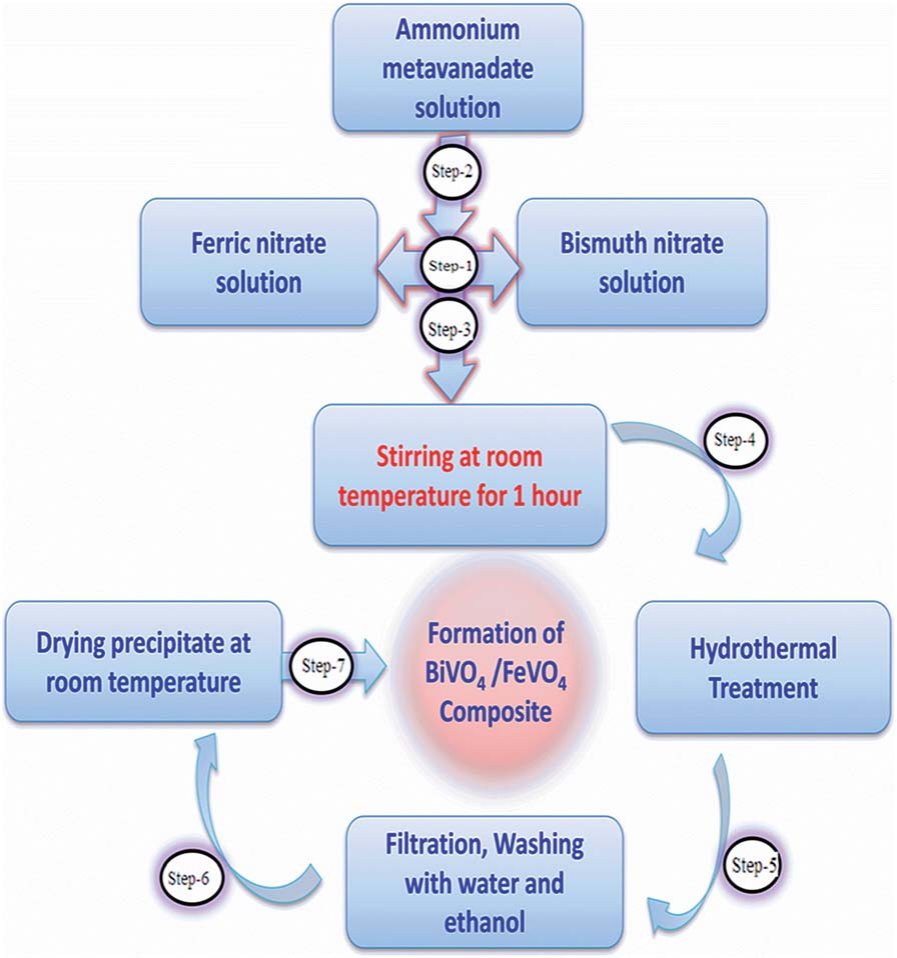
Flow chart of the hydrothermal method for the preparation of BiVO_4_/FeVO_4_.

All of the materials were of analytical grade and used without any purification. The heterojunction composite BiVO_4_/FeVO_4_ was fabricated *via* a hydrothermal method. Firstly, 1 mmol Bi(NO_3_)_3_·5H_2_O and 1 mmol Fe(NO_3_)_3_·9H_2_O were dissolved in 60 mL distilled water, then 2 mmol of NH_4_VO_3_ was poured into the solution and vigorous stirring was carried out for 1 h at ambient temperature. The pH was adjusted to around 8 using NaOH, and the solution was transferred into a 100 mL Teflon-lined stainless autoclave, which was heated at 180 °C for 24 h. After cooling the autoclave, the precipitate was centrifuged and washed many times with ethanol and distilled water. It was then dried at 80 °C.^30,31^ To check the effects of various concentrations on the crystallinity, morphology, photocatalytic and electrochemical sensing properties, BiVO_4_/FeVO_4_ heterojunctions were prepared in different molar ratios while keeping all other parameters the same, resulting in gradual color changes from blackish to yellowish as the BiVO_4_ concentration increased. The flow chart of the preparation method is shown in Fig. 1.

### Characterization of BiVO_4_/FeVO_4_

A Rigaku 2500 X-ray diffractometer was used to analyze the crystallinity, phase and particle size of the synthesized nanoparticles. The morphological features and topography were characterized using field emission scanning electron microscopy, on a JEOL-7001F working at an operating voltage of 20 kV. The elemental analysis of the nanoparticles was examined using energy-dispersive X-ray spectroscopy (EDX), with an FESEM microscope attached to an XPS spectrometer (PHI 5000 Versa Probe). The absorbance spectra of organic CV dye were measured using a PerkinElmer λ-35 UV-vis spectrophotometer. The porosity and Brunauer–Emmett–Teller (BET) surface areas of the products were evaluated by a multi-point BET method using adsorption data. FT-IR measurements were obtained on a Nicolet Magna-550 spectrometer. Photoluminescence (PL) and Raman analysis were conducted on Horiba Scientific Fluoromax-4 spectrofluorometer. Electronic Paramagnetic Resonance (EPR) analysis was performed on an ESR-JES-FA 2010 spectrometer.

### Photocatalytic activity

To check the photocatalytic responses of powdered BiVO_4_/FeVO_4_, samples were illuminated using a UV-visible light xenon lamp with an accumulative intensity of 300 W for degrading CV dye, which was 25 cm away from the lamp. For studying the kinetics, dye solutions with fixed concentrations (150 mL) at neutral pH were prepared and 0.1 g of the prepared BiVO_4_/FeVO_4_ photocatalyst was mixed into each separate solution. The pH was maintained by adding HNO_3_ and NaOH, and then each prepared solution was stirred for 15 min in the dark and finally centrifuged at 10 000 rpm for 5 min. To avoid any thermal degradation and to keep the temperature at 0 °C, nitrogen cooling was used throughout the experiment.^32^ The degradation phenomenon of the dye was analyzed using a PerkinElmer λ-35 UV-vis spectrometer in the spectral range of 400–800 nm.

### Sensor measurements

The electrochemical evaluation was carried using a model Chi760D electrochemical work station, and a three-electrode scheme. A glassy carbon electrode (GCE) was applied in this work for electrode adjustment. BiVO_4_/FeVO_4_ (0.5 mg) was first dispersed in 1 mL distilled water. Then, 10 μL of nanomaterial was put on the GC electrodes and dehydrated in the oven at 80 °C for 2 min, after which 2–3 μL of 5% Nafion was drop-casted onto the electrodes and dried out. Each solution for sensor measurements comprised distilled water, and the required concentrations were made up using suitable dilutions from the respective stock solutions. The BiVO_4_/FeVO_4_ modified glassy electrode was applied as the working electrode, a carbon glassy rod as the counter electrode and Ag/AgCl electrode as the reference electrode. Before the electrochemical measurements, each solution was degassed using nitrogen gas for 4–5 min. Electrochemical impedance spectroscopy (EIS) was carried out at an amplitude of 0.01 V in the frequency range of 0.1–4000 Hz. Cyclic voltammetry measurements were recorded in the potential range of −1.0 V to +1.0 V at a scan rate of 0.05 V s^−1^ in a mixed solution of 0.1 M H_3_PO_4_, Li_2_SO_4_, NaSO_4_ and NaOH with ascorbic acid at 0.05 mM concentration. All measurements were executed at room temperature.

## Results and discussion

**Fig. 2:**
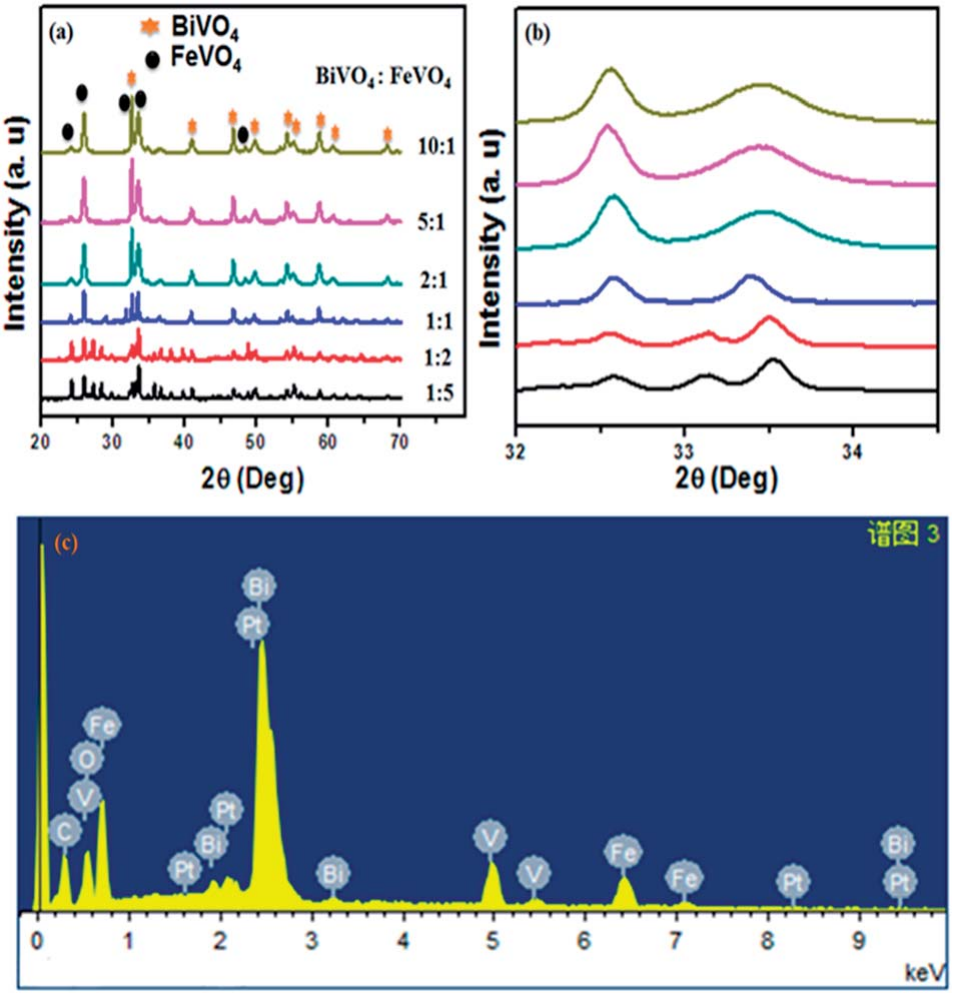
(a) XRD spectrum of BiVO_4_/FeVO_4_ composite heterojunction photocatalysts at different molar ratios of 1 : 5, 1 : 2, 1 : 1, 2 : 1, 5 : 1 and 10 : 1. (b) Peak shifts in the XRD spectrum of BiVO_4_/FeVO_4_ due to increasing concentration of BiVO_4_. (c) EDS analysis of BiVO_4_/FeVO_4_ composite at a molar ratio of 2 : 1.

Fig. 2a shows the XRD diffraction pattern of the as-prepared BiVO_4_/FeVO_4_ nanophotocatalysts at different concentration ratios for phase structures. The XRD diffraction peaks of BiVO_4_/FeVO_4_ are in agreement with pure BiVO_4_ (JCPD card 85-1730)^33^ and FeVO_4_ (JCPD card 24-0541). The patterns of the BiVO_4_/FeVO_4_ heterojunction photocatalyst compounds display typical diffraction peaks from both BiVO_4_ and FeVO_4_ distinct phases, verifying that the BiVO_4_/FeVO_4_ nanocomposites at different molar ratios were prepared successfully by the autoclave hydrothermal process, as shown in Fig. 2a. The intense and edged peaks of BiVO_4_ suggest a larger crystallite size, while the small peaks of FeVO_4_ indicate a small particle size. There is a shift in the peaks (Fig. 2b) first towards the left when the ratio is 2 : 1, and then towards the right as the ratio increases from 5 : 1 to 10 : 1. The color also changes gradually into yellowish as the BiVO_4_ concentration is increased. It can be clearly observed that the broadening of BiVO_4_ peaks takes place and the intensity of the FeVO_4_ peaks declines. Here, the optimum conditions were revealed for making BiVO_4_/FeVO_4_ (2 : 1) at which high intensity peaks are found, as indicated in Fig. 2a. From the EDS analysis shown in Fig. 2c, it is observed that the elements vanadium, iron, bismuth and oxygen are homogeneously spread throughout the composite, and the presence of carbon and platinum peaks are due to carbon tape and platinum coating. Aside from this, no impurities were detected within the detection limit.

**Fig. 3:**
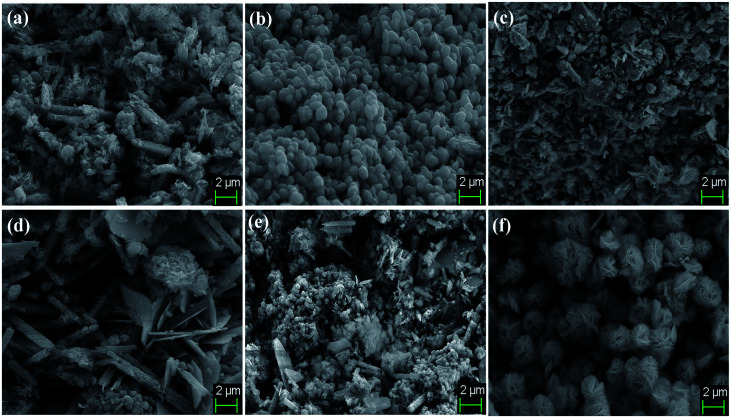
SEM images of BiVO_4_/FeVO_4_ composite heterojunction photocatalysts at different molar ratios of (a) 1 : 5, (b) 1 : 2, (c) 1 : 1, (d) 2 : 1, (e) 5 : 1 and (f) 10 : 1.

The surface topography and morphological analysis of the BiVO_4_/FeVO_4_ nanocomposites were characterized using field emission scanning electron microscopy, on a JEOL-7001F. SEM images of the composites at different ratios (1 : 5, 1 : 2, 1 : 1, 2 : 1, 5 : 1 and 10 : 1) are illustrated in Fig. 3a–f. It was noticed that at higher concentrations of FeVO_4_, small sized nanocomposites developed, as shown in Fig. 3a and b. When the BiVO_4_/FeVO_4_ ratio was 1 : 5, rod and particle shaped nanostructures formed, while at a ratio of 1 : 2, polyhedron and particle shaped nanostructures formed. In the opposite case, upon increasing the molar ratio of BiVO_4_ in the BiVO_4_/FeVO_4_ nanocomposite, large crystallite sizes were found, as shown in Fig. 3d–f. In Fig. 3d the BiVO_4_/FeVO_4_ nanocomposite at a molar ratio of 2 : 1 comprises rods and plates, and some plates are linked with each other to form chains that look like flower type structures. At a molar ratio of 5 : 1, the SEM image captured shows rod and plate-like structures, in which there are more agglomerations of plates than at the previous concentration ratio. The SEM image in Fig. 3f of the BiVO_4_/FeVO_4_ nanocomposite at a molar ratio 10 : 1 shows flowers and just a few plate structures are found.

The SEM morphologies of pure BiVO_4_ and FeVO_4_ are given in Fig. S1 (ESI).† The formation and conversion from particles to nanorods and flower shape morphologies are totally dependent on the experimental conditions, reaction mechanisms and material compositions. In our experimental results, rod and particle shaped nanostructure formation occurs when the FeVO_4_ concentration is higher than that of BiVO_4_. Contrarily, as the BiVO_4_ concentration increases, plate-like crystallites begin to appear, ultimately forming flower shaped nanostructures. These morphological changes take place due to differences in structure, atomic radii, crystal orientation and reactions occurring during the hydrothermal synthesis. Thus, our experimental results validate that nanorod and particle conversion into nanoplate flower-like morphologies takes place as the ratio of BiVO_4_ and FeVO_4_ is changed during the synthesis of the BiVO_4_/FeVO_4_ nanocomposites, as shown in the SEM image (Fig. 3).

**Fig. 4:**
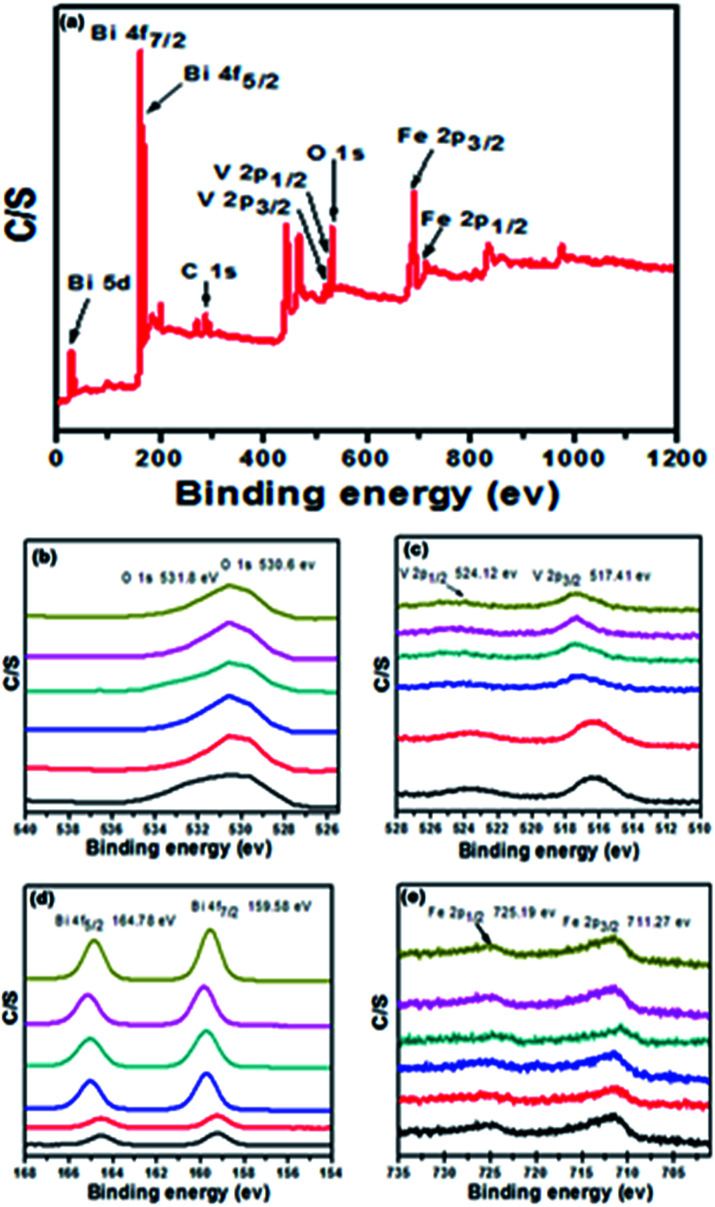
XPS of BiVO_4_/FeVO_4_ nanocomposite at different molar ratios of BiVO_4_ and FeVO_4_.

X-ray photoelectron spectroscopy (XPS) is a surface analysis method for investigating the composition and chemical states of the constituents. In order to validate further, elemental analysis was carried out by performing XPS characterization. Fig. 4a shows the typical X-ray photoelectron spectrum of the BiVO_4_/FeVO_4_ nanocomposite, which is composed of O, Bi, V, and Fe. In the XPS analysis of the 2 : 1 BiVO_4_/FeVO_4_ nanocomposite, two peaks of oxygen at 1s are located at 530.6 and 531.8 eV^34,35^ in Fig. 4b, showing the two different peaks of O_2_ in the experiment. Two peaks of V 2p_3/2_ at 517.41 eV and V 2p_1/2_ at 524.12 eV were noticed, as shown in Fig. 4c. Fig. 4d highlights the XPS peaks of Bi 4f_7/2_ and Bi 4f_5/2_, which are found at 159.44 eV and 164.68 eV.^36^ In Fig. 4e, the Fe 2p spectrum comprises two leading peaks (2p_1/2_ and 2p_3/2_) with many sub-peaks, with the main peaks positioned at 711.27 and 725.19 eV.^37^ A peak shift was found similar to that observed in the XRD analysis, which may be attributed to morphology effects or the increasing concentration of BiVO_4_. From the above XRD and XPS detailed spectra, the formation of the BiVO_4_/FeVO_4_ heterojunction photocatalysts can be confirmed.

**Fig. 5:**
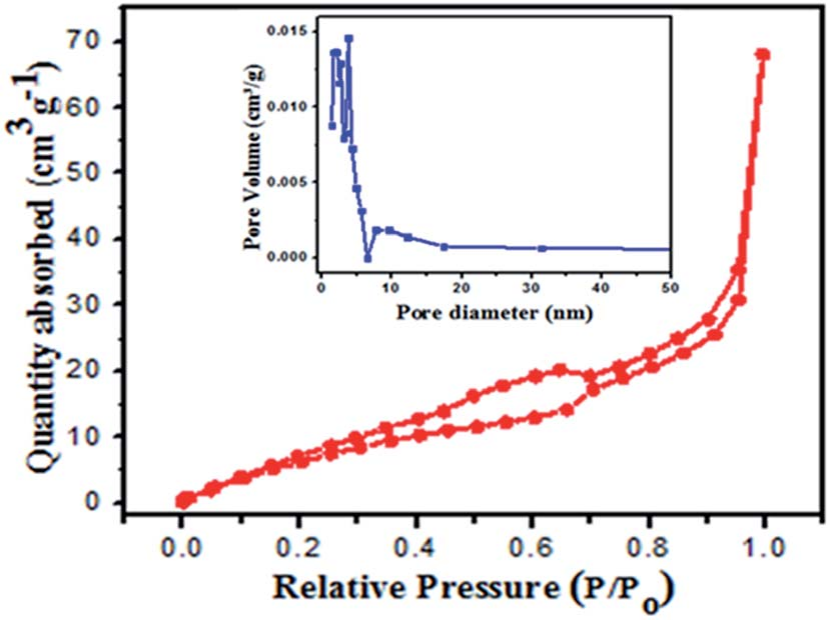
BET surface area (inset pore diameter) of the 2 : 1 BiVO_4_/FeVO_4_ composite at 77.35 K.

The N_2_ sorption isotherms of the 2 : 1 nanocomposite photocatalyst BiVO_4_/FeVO_4_ were produced and examined. The Brunauer–Emmett–Teller surface area was found to be 70.147 cm^2^ g^−1^. The pore size was estimated from the desorption isotherms, using the Barret–Joyner–Halender (BJH) method.^38,39^ The evaluated pore volume and average pore diameter were found to be 0.124 cm^3^ g^−1^ and 3.798 nm, respectively, as shown in Fig. 5. The adsorption curve is a type III curve presenting the hysteresis of H_2_ and H_3_ types, which associates slit shape capillaries with large and narrow short plate-like aggregates of particles, resulting in a lamellar pore structure and slit shaped pores.^40^ The lamellar wedge shaped pore construction clearly indicates the presence of mesopores within the structure.

The Brunauer–Emmett–Teller surface areas of the BiVO_4_/FeVO_4_ composites at molar ratios of 1 : 5, 1 : 2, 1 : 1, 5 : 1 and 10 : 1 were 136.188, 6.019, 7.097, 9.325 and 14.679 cm^2^ g^−1^, the pore volumes were 0.213, 0.010, 0.012, 0.013 and 0.025 cm^3^ g^−1^, and the pore diameters were 1.682, 1.932, 1.682, 1.937 and 2.181 nm, respectively, as shown in Fig. S2 (ESI).†

**Fig. 6:**
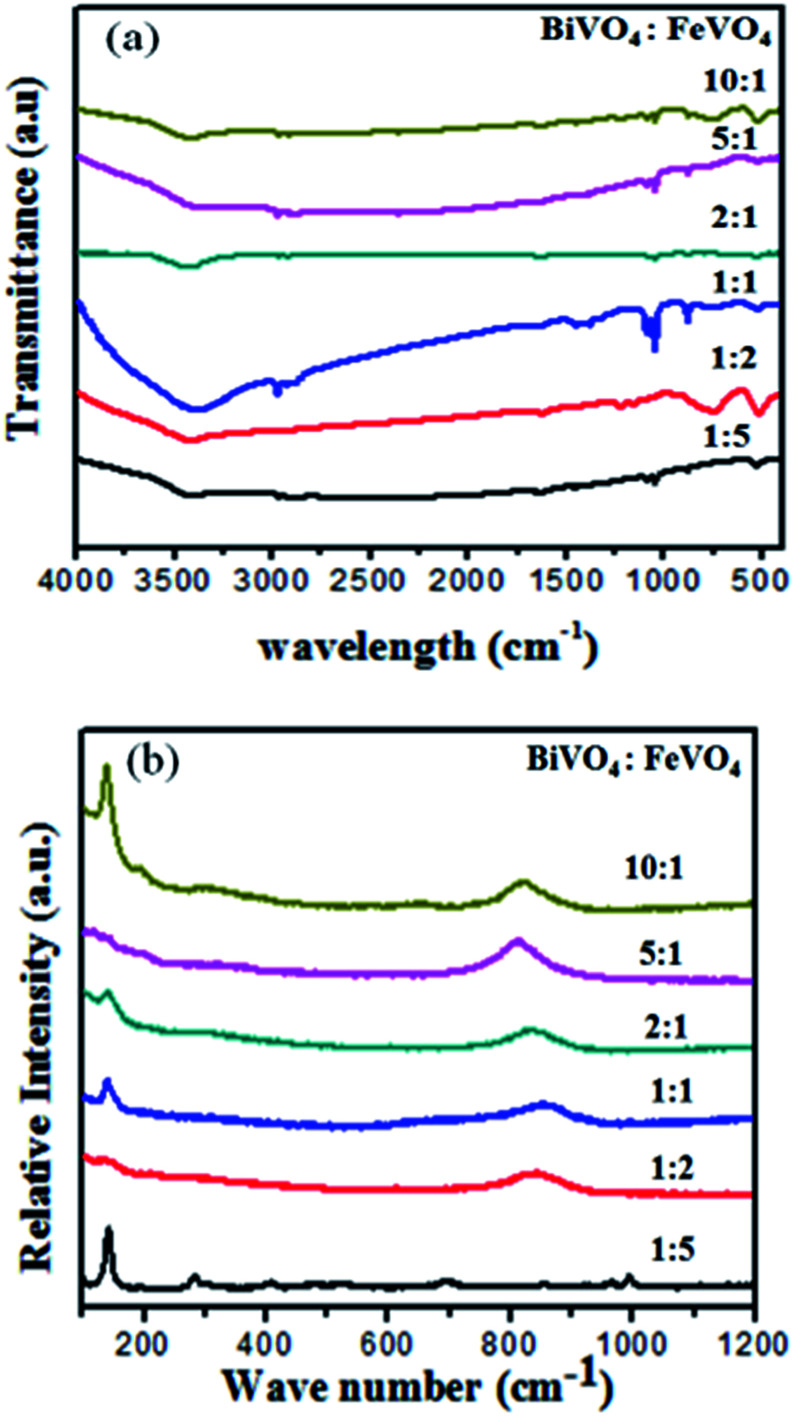
(a) FT-IR spectra and (b) Raman spectra of the BiVO_4_/FeVO_4_ heterogeneous composites prepared with different molar ratios.

Fig. 6a shows the FT-IR spectra of the BiVO_4_/FeVO_4_ heterogeneous composites prepared under different molar ratios, where the absorption from 3000 to 3600 cm^−1^ is due to the stretching vibration of OH of absorbed water. The absorption at 1631 cm^−1^ is due to absorbed water molecule bending vibration. The band at 512 cm^−1^ is due to V–O–V deformation caused by the V–O stretching mode. The peaks at 741, 837, 915 and 1052 cm^−1^ correspond to V=O and V–O–V joined vibrations and the stretching of the short vanadyl bond, while the Bi–O bending and asymmetric vibrations appear at 474 cm^−1^ and 1362 cm^−1^, respectively.^41–45^

Raman analysis is a useful tool for providing structural information and is also a sensitive method for investigating crystallization, local structure and the electronic dimensions of materials. In Fig. 6b, Raman bands at around 198, 333, 367, 640 and 836 cm^−1^ corresponding to BiVO_4_ were observed for all samples, while bands at around 139, 300, 354, 655, 962 and 996 cm^−1^ represent FeVO_4_ in the samples.^46–48^ A shift in the peaks was observed as the molar ratio changed. At higher concentrations of BiVO_4_, the peaks were broader and covered the FeVO_4_ peaks, as observed in the XRD and XPS analysis, which also confirms and supports our previous results.

### Photocatalytic activity of crystal violet solution

To determine the photocatalytic activity of FeVO_4_, BiVO_4_ and the BiVO_4_/FeVO_4_ nanophotocatalysts, the degradation rate of CV dye was investigated in water under visible radiation from 400 < *λ* < 800 nm. The chemical formula of CV is C_25_H_30_ClN_3_, and its chemical structure with its adsorption spectrum is shown in Fig. S3 (ESI).† The photocatalytic response of the BiVO_4_/FeVO_4_ photocatalysts is shown in Fig. S4 (ESI).† It is noticed that the photocatalytic action of 2 : 1 BiVO_4_/FeVO_4_ is significantly higher compared to the other samples. It degraded 99.1% of CV dye within 60 min under visible radiation illumination. It is clear that in both cases of either increasing the FeVO_4_ or BiVO_4_ molar ratio, the photocatalytic activity is reduced. However, in the case of a higher concentration of FeVO_4_, the photocatalytic activity first increases and then reduces.

When the molar ratio of BiVO_4_/FeVO_4_ is 1 : 5, the photocatalytic degradation efficiency of CV is 71% in 60 min. Perhaps the most appropriate BiVO_4_/FeVO_4_ molar ratio to form a nanocomposite heterojunction photocatalyst is 2 : 1. The 2 : 1 BiVO_4_/FeVO_4_ heterojunction photocatalyst facilitates effective electron–hole separation, reduces the recombination rate of charges and enhances the absorption of visible light. According to the above results and discussion, a higher concentration of BiVO_4_ or FeVO_4_ leads to reduced photocatalytic activity, which may be attributed to some particles of BiVO_4_ or FeVO_4_ that cannot be effectively incorporated into the composite photocatalyst. In this study, the 2 : 1 BiVO_4_/FeVO_4_ composite exhibited the highest degradation efficiency and was therefore determined to have the optimal ratio.

**Fig. 7:**
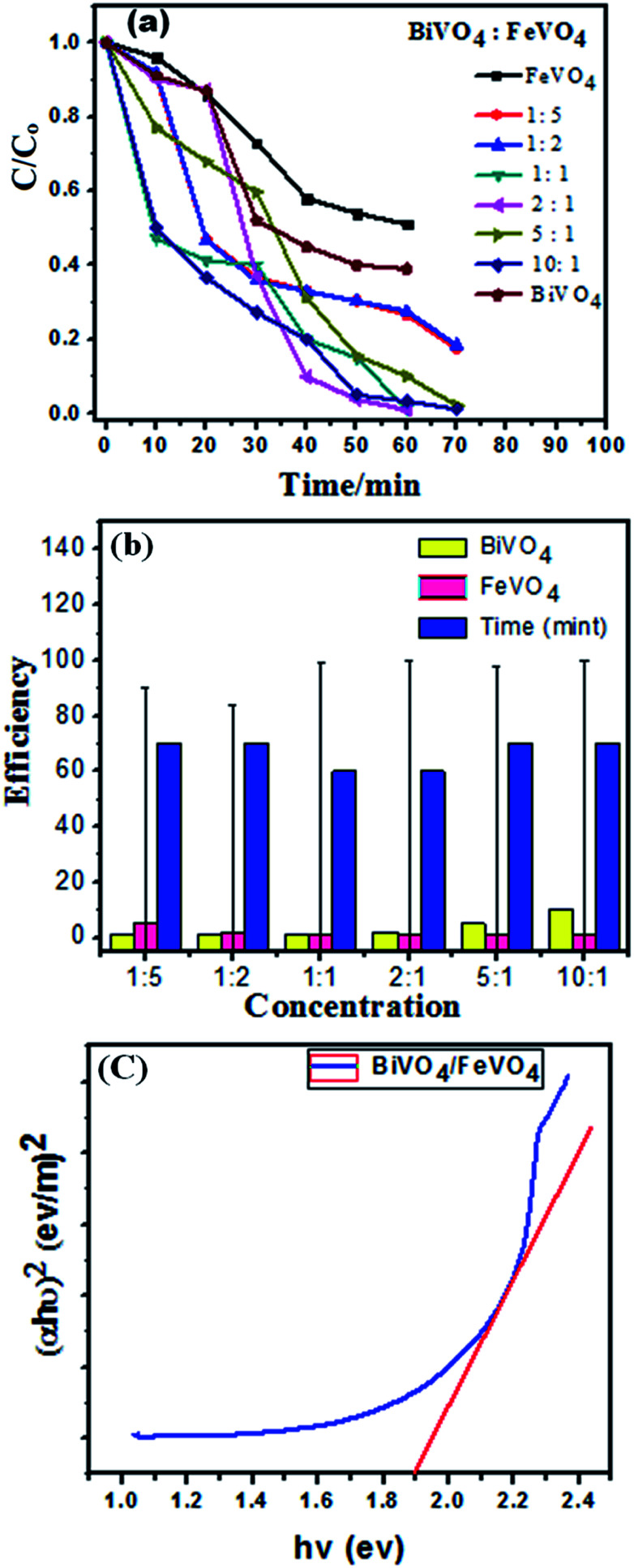
(a) Concentration changes of CV dye as a function of irradiation time using FeVO_4,_ BiVO_4_ and BiVO_4_/FeVO_4_ at molar ratios of 1 : 5, 1 : 2, 1 : 1, 2 : 1, 5 : 1 and 10 : 1. (b) Degradation error bar profile of CV over BiVO_4_/FeVO_4_ at different molar ratios as a function of time. (c) Tauc plot for BiVO_4_/FeVO_4_ composite with a 2 : 1 ratio.

The degradation efficiency is determined by following the equation:

1 Degradation efficiency% = (*C*_o_ − *C*_*t*_)/*C*_o_

where *C*_o_ is the initial concentration at the time *t*_0_, and *C*_*t*_ is the concentration at any time *t*. Fig. 7a and b show the degradation efficiency at the same catalyst dose. The energy band gap is an important parameter in semiconductor materials for evaluating their properties and applications, so UV-vis spectra of the prepared BiVO_4_/FeVO_4_ nanocomposite was calculated using the Tauc plot relation,^49^ as below:

2 (*αhν*) = *A*(*hν* − *E*_g_)^*n*^

Here, *α* is the absorption coefficient, *A* is a constant and *n* = 1/2 for a direct band gap material.

**Fig. 8:**
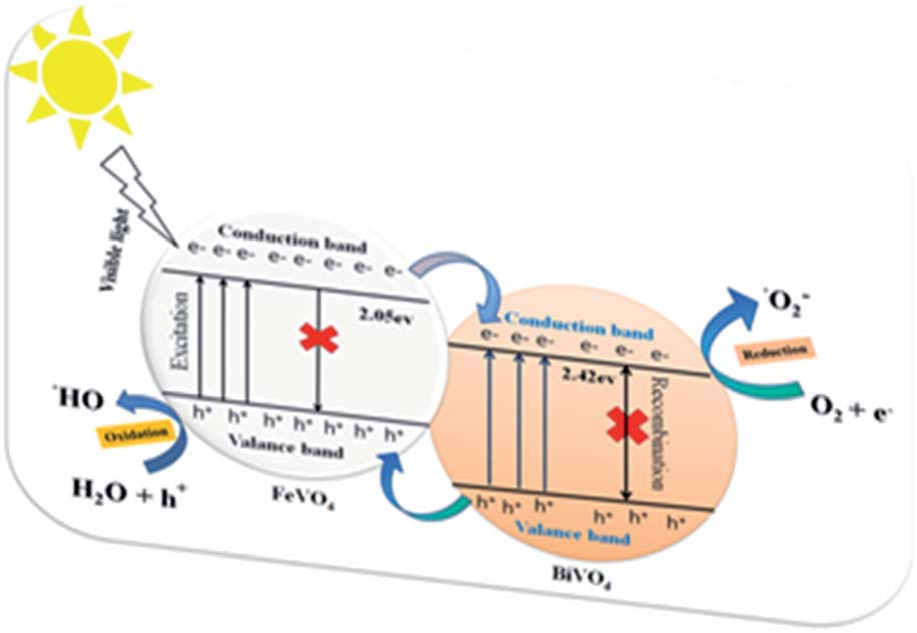
Reaction mechanism of CV photodegradation over the BiVO_4_/FeVO_4_ composite under visible light.

The energy band gap (*E*_g_) of the BiVO_4_/FeVO_4_ compound was calculated by interpolating the linear portion of the plot of (*αhν*)^2^*versus hν* to the energy axis, as shown in Fig. 7c. The *E*_g_ value came out to be 1.9 eV for BiVO_4_/FeVO_4_, which is closely matched to the described values. The photocatalytic mechanism for the degradation of CV dye solution by BiVO_4_/FeVO_4_ under visible light is illustrated in Fig. 8 and can be represented by the following equations:

3 BiVO_4_/FeVO_4_ + *hν* → e^−^ + h^+^ + BiVO_4_/FeVO_4_

Oxidation occurs at the FeVO_4_ surface:

4 h^+^ + H_2_O → ˙OH + H^+^

5 2h^+^ + 2H_2_O → 2H^+^ + H_2_O_2_

Reduction reaction occurs at BiVO_4_:

6 e^−^ + O_2_ → ˙O_2_^−^

When visible light falls on the solution containing CV dye and the BiVO_4_/FeVO_4_ photocatalyst, the electrons move fast from FeVO_4_ to BiVO_4_, while the holes of BiVO_4_ move to the valence band (VB) of FeVO_4_. An oxidation reaction occurs at the VB of FeVO_4_, where positive holes react with water to form hydroxyl radicals (˙OH), while a reduction reaction occurs at the conduction band (CB) of BiVO_4_, where negative electrons (e^−^) produce superoxide radicals (˙O_2_^−^) by reacting with dissolved oxygen. The superoxide radicals and hydroxide radicals from both ends oxidize the toxic dye C_25_H_30_ClN_3_ molecules and decompose them into harmless or non-toxic molecules with CO_2_, H_2_O and NO_3_ byproducts, as eqn (7) indicates below.

7 C_25_H_30_ClN_3_ + (˙OH, ˙O_2_^−^) → CO_2_ + H_2_O + NO_3_ + other intermediates

During the photocatalysis process, various primary active species including photogenerated holes, singlet oxygen atoms, hydroxyl radicals and superoxide radicals could be created during the UV-visible degradation process.^50–52^ According to previous studies, in the presence of N_2_ and radical scavengers, ˙OH and ˙O_2_^−^ are the two main active species in the entire process.^5,45,50,52–56^ From previous results, the dominant active oxygen species generated in direct oxidation and photocatalytic reactions are ^1^O_2_ and ˙OH radicals.^32,50,57,58^ On the basis of the above studies, the probability of forming ˙OH should be much lower than that for ˙O_2_^−^; however, the hydroxyl radical is an extremely strong oxidizing agent, which takes the degradation process to either partial or complete mineralization of various organic contaminations. The photocatalytic degradation of CV dye by pure FeVO_4_, pure BiVO_4_ and the BiVO_4_/FeVO_4_ heterojunction nanophotocatalysts at molar ratios of 1 : 5, 1 : 2, 1 : 1, 2 : 1, 5 : 1 and 10 : 1 was performed and absorbance spectra are provided in the ESI (Fig. S4†).

**Fig. 9:**
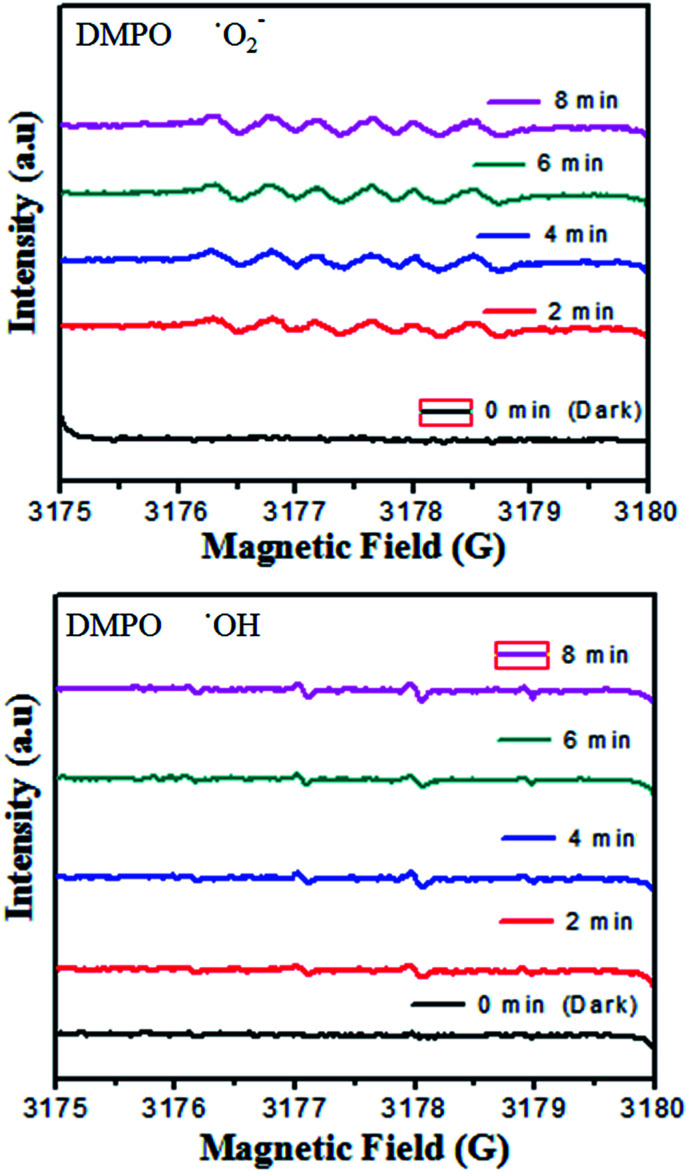
DMPO spin trapping EPR spectra for DMPO-˙O_2_^−^ and DMPO-˙OH under visible light irradiation with the BiVO_4_/FeVO_4_ photocatalyst.

On the basis of the aforementioned studies,^5,32,52,56,58^ once the electrons enter the conduction band of BiVO_4_, it induces the production of oxygen, which causes decomposition of CV dye. Hydroxyl radicals are also produced by the reaction of ˙O_2_^−^ radicals with H^+^ ions and h^+^ holes with OH^−^ ions or H_2_O. No electron paramagnetic resonance (EPR) signal was noticed when the reaction was executed in the dark. However, the signals with intensities corresponding to the characteristic peaks of DMPO-˙OH and DMPO-˙O_2_^−^ adducts were observed during the reaction process in the EPR experiment. In Fig. 9, not only are the six characteristic peaks of the DMPO-˙O_2_^−^ observed, but the four characteristic peaks of the DMPO-˙OH radical (1 : 2 : 2 : 1; quartet pattern) are also observed by irradiation of the BiVO_4_/FeVO_4_ heterojunction nanocomposite solution under visible light. The decay of CV dye by the generated oxidant species is represented by eqn (8) and (9).

8 CV + ˙O_2_^−^ → decomposed compounds

9 CV + ˙OH → decomposed compounds

The effects of catalyst dose, dye concentration, stability and recyclability factors were also investigated and the results are provided in the ESI (Fig. S5†). In the absorption spectra of CV solution using BiVO_4_/FeVO_4_ at different doses of 0, 2.5 and 5 mg/10 mL, it is observed that the rate of degradation decreases as the dose of the BiVO_4_/FeVO_4_ decreases by 5 mg/10 mL to 0 mg/10 mL in CV solutions.

This implies that the amount of BiVO_4_/FeVO_4_ catalyst has a significant effect on the photocatalytic reaction for the decomposition of CV organic dye. Dye concentration effect was evaluated by taking different initial concentrations of CV dye (10–50 mg L^−1^) and the results are shown in Fig. S5 (ESI).† The color removal efficiency decreases as the CV concentration is increased; this is due to the fact that the increasing concentration of CV prevents light penetration into the solution. Secondly, the number of CV molecules absorbed on the catalyst surface is increased, while the numbers of OH and O radicals remain the same under specific conditions. Furthermore, the stability and recyclability of the BiVO_4_/FeVO_4_ heterogeneous photocatalyst were also investigated, as shown in Fig. S5 (ESI).† The photocatalytic activity was evaluated three times with the BiVO_4_/FeVO_4_ photocatalyst and the sample showed superb stability as well as recyclability.

**Fig. 10:**
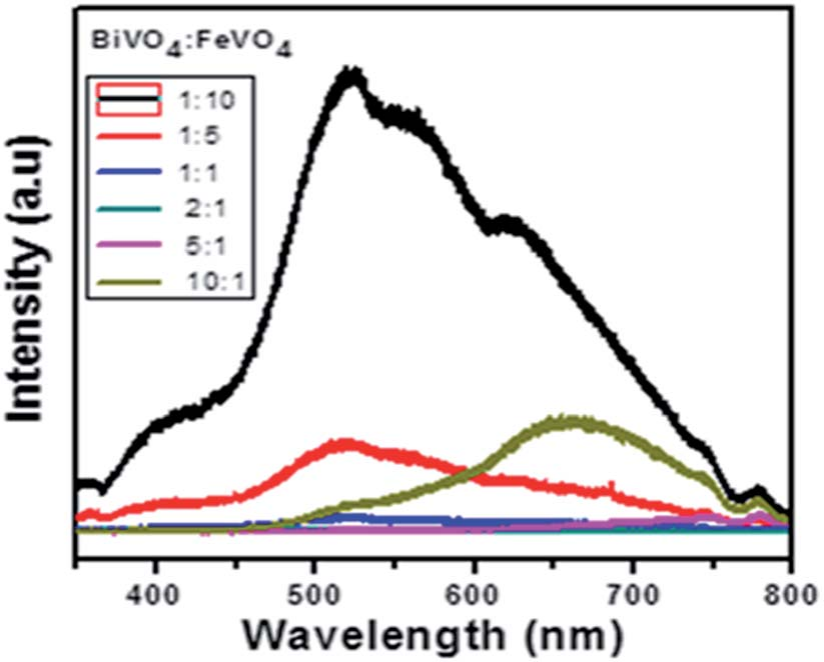
PL spectra of the BiVO_4_/FeVO_4_ composites at different concentrations.

The photoluminescence spectra exhibit the recombination rate and electron–hole separation within the photocatalysts. A higher PL intensity reveals a higher degree of electron–hole recombination, which reduces the photocatalytic degradation efficiency.^59^ A lower PL peak intensity indicates reduced recombination, which results in efficient charge transfer over the catalyst surface in a semiconductor.^60,61^ So, the low recombination allows superior dye degradation, which is consistent with the photocatalytic results shown in Fig. 10.

**Fig. 11:**
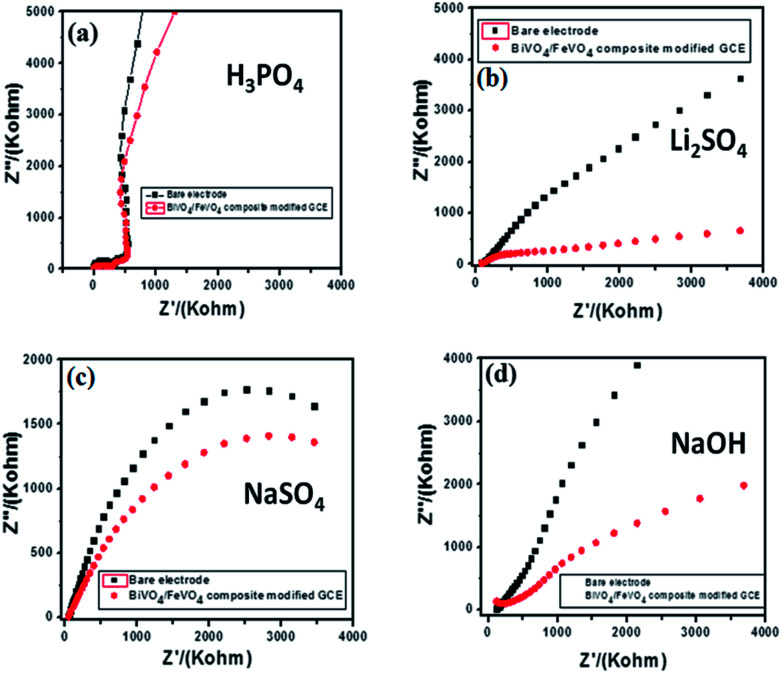
Electrochemical impedance spectroscopy of bare and modified BiVO_4_/FeVO_4_ nanocomposite GCEs in different electrolytes: (a) 0.1 M H_3_PO_4_, (b) 0.1 M Li_2_SO_4_, (c) 0.1 M NaSO_4_ and (d) 0.1 M NaOH.

**Fig. 12:**
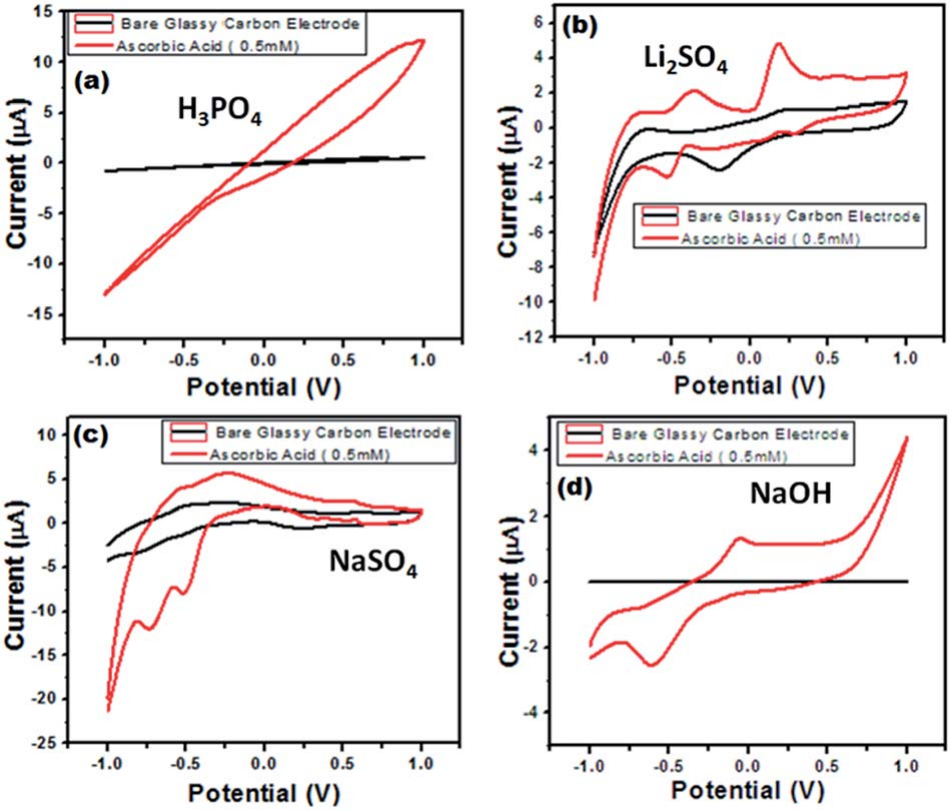
Cyclic voltammograms of the BiVO_4_/FeVO_4_ modified GCEs in (a) 0.1 M H_3_PO_4_, (b) 0.1 M Li_2_SO_4_, (c) 0.1 M NaSO_4_ and (d) 0.1 M NaOH solution in the absence and presence of 0.5 mM ascorbic acid. Scan rate = 50 mV s^−1^.

Electrochemical impedance spectroscopy is a versatile tool for measuring the conductivity, surface analysis and electron transfer.^62^ It was noticed that in all electrolytes, the impedance spectrum is either a semicircle or nearly a circle at higher ac frequency and a line at low modulation ac frequency, as shown in Fig. 11. Moreover, in the modified BiVO_4_/FeVO_4_ nanocomposite GCE, the diameter of the Nyquist circle decreases as compared to that of the bare GCE, indicating that the BiVO_4_/FeVO_4_ nanocomposite exhibits much higher electron transfer on the surface and superior electrochemical activity, and as a result, the resistance decreases and improves the electron transfer process. Cyclic voltammetry was used to assess the detection of ascorbic acid analyte by the BiVO_4_/FeVO_4_ nanocomposite material. Cyclic voltammetry showed the response of the bare electrode and glassy carbon modified electrode both in the presence and absence of 0.5 mM ascorbic acid at a scan rate of 50 mV s^−1^ at −1 to 1 V s^−1^ as shown in Fig. 12a. The bare electrode and modified glassy carbon electrode in H_3_PO_4_ solution did not show any oxidation peak both in the absence and presence of ascorbic acid. However, an enhanced current was observed, which shows that the semiconductor material has the potential to change the current intensity.

In LiSO_4_ solution (Fig. 12b) the bare GCE gives one oxidation peak and one reduction peak located at 0.20 V and −0.20 V, respectively. Meanwhile, the BiVO_4_/FeVO_4_ nanocomposite GCE response shows two anodic peaks (cvp1 = −0.36 V, cvp2 = −0.17 V) and two cathodic peaks (cvp1′ = 0.52 V, cvp2′ = 0.19 V) as well as an increase in the current intensity. In the case of NaSO_4_ solution (Fig. 12c), the bare electrode did not show any oxidation and reduction but the modified BiVO_4_/FeVO_4_ nanocomposite GCE showed two anodic peaks (cvp1 = −0.49 V, cvp2 = −0.71 V). In the case of the basic solution (NaOH), the bare GCE did not give any response, as shown in Fig. 12d. However, the modified BiVO_4_/FeVO_4_ nanocomposite GCE showed a change in current intensity, as well as one reduction peak (cvp = −0.05 V) and one oxidation peak located at cvp = −0.59 V.

**Fig. 13:**
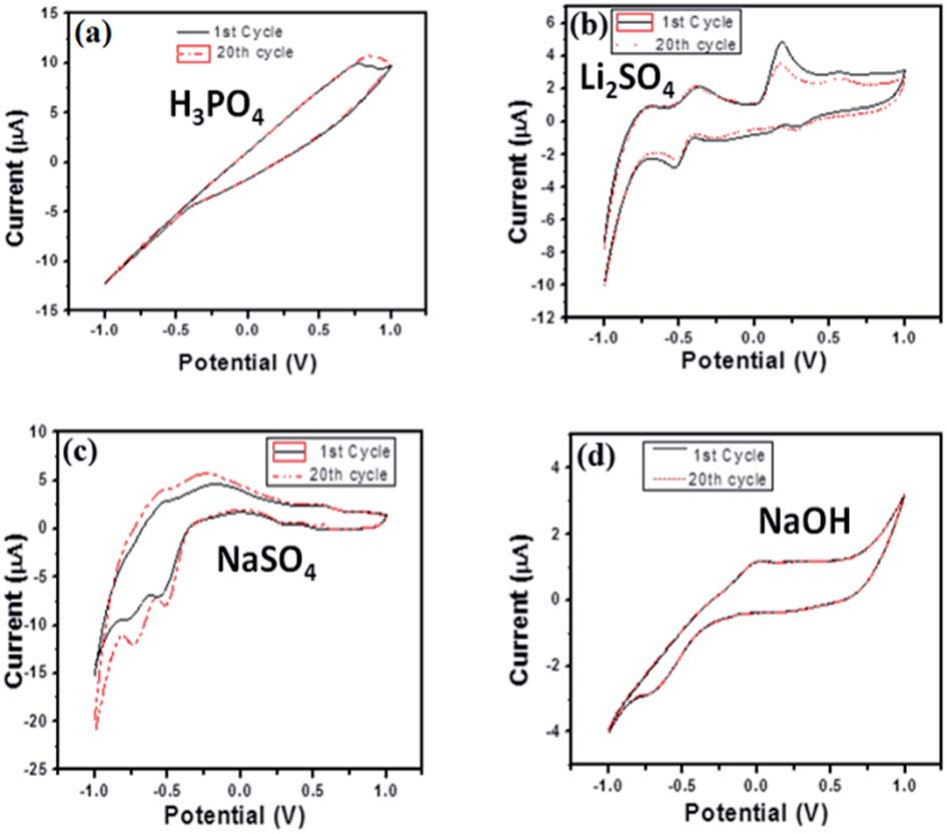
Cyclic voltammograms of the BiVO_4_/FeVO_4_ modified GCE in mixed (a) 0.1 M H_3_PO_4_, (b) 0.1 M Li_2_SO_4_, (c) 0.1 M NaSO_4_, (d) 0.1 M NaOH and ascorbic acid (0.5 mM) solutions. Scan rate = 50 mV s^−1^. Recycling for 1^st^ and 20^th^ time.

For long-term stability confirmation we stored the modified BiVO_4_/FeVO_4_ nanocomposite GCE for one month at room temperature and used it for sensing ascorbic acid. It was found that it detects ascorbic acid without any decrease in current. Furthermore, we checked its reproducibility after twenty measurements, and it was found to vary slightly relative to the standard deviation of the original values, as indicated in Fig. 13. Thus, the modified BiVO_4_/FeVO_4_ nanocomposite GCE exhibits excellent stability and reproducibility for the electrochemical determination of ascorbic acid.

## Conclusions

BiVO_4_/FeVO_4_ nanocomposites were synthesized *via* an autoclave hydrothermal method using bismuth nitrate dehydrate (Bi(NO_3_)_3_·5H_2_O) and iron nitrate dehydrate (Fe(NO_3_)_3_·9H_2_O) as bismuth and ferric ion sources and ammonia metavanadate NH_4_VO_3_ as a vanadium ion source, at different BiVO_4_ and FeVO_4_ molar ratios. The prepared nanocomposites were characterized using X-ray diffraction (XRD), scanning electron microscopy (SEM), energy dispersive X-ray spectroscopy (EDX), X-ray photoelectron spectroscopy (XPS), the Brunauer–Emmett–Teller (BET) method, Fourier transform infrared (FT-IR) spectroscopy, Raman spectroscopy, photoluminescence (Pl), electron paramagnetic resonance, cyclic voltammetry and electrochemical impendence spectroscopy (EIS). The BiVO_4_/FeVO_4_ nanocomposites were investigated for their activity as nanophotocatalysts by the photocatalytic degradation of crystal violet (CV) dye under visible light irradiation. The as-synthesized heterojunction photocatalysts exhibited good photocatalytic activity, especially BiVO_4_/FeVO_4_ at a molar ratio of 2 : 1, which showed superior degradation efficiency owing to the higher specific surface area of 70.147 cm^2^ g^−1^ and pore size of 3.798 nm. The material was also studied for the electrochemical detection of an important analyte, ascorbic acid, and it showed good results. This work reveals the potential of BiVO_4_/FeVO_4_ nanocomposites for applications in environmental science as well as in biosensor fields.

## Conflicts of interest

The authors declare no conflict of interest.

## Supplementary Material

RA-008-C8RA03890B-s001
