# Supplementary material for: Visible light assisted photocatalytic degradation of crystal violet dye and electrochemical detection of ascorbic acid using a BiVO4/FeVO4 heterojunction composite
Source: RSC Adv. 2018 Jun 27;8(42):23489–98. doi: 10.1039/c8ra03890b (PMC9081775; doi:10.1039/c8ra03890b)
Supplement: RA-008-C8RA03890B-s001 [file RA-008-C8RA03890B-s001.pdf]

Electronic Supplementary Material (ESI) for RSC Advances.
This journal is © The Royal Society of Chemistry 2018

Visible light assisted photocatalytic degradation of crystal violet dye and electrochemical detection of ascorbic acid using $BiVO_4/FeVO_4$ heterojunction composite

Muhammad Munir Sajid [a, b, c], Sadaf Bashir Khan[b], Naveed Akthar Shad[a,c], Nasir Amin[a] and Zhengjun Zhang[*d]

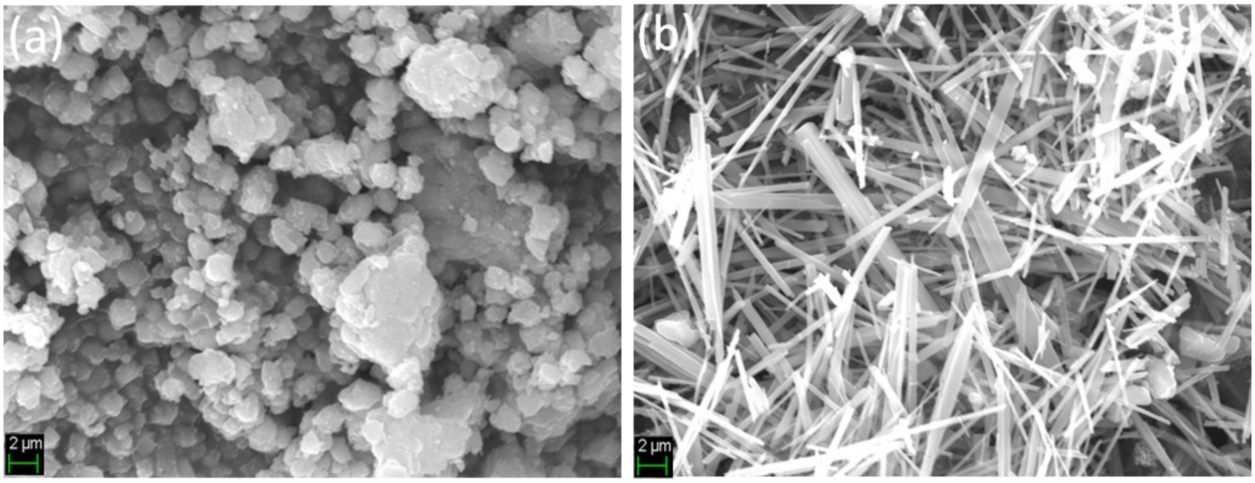

Figure. S1 Show the SEM images of pure (a) BiVO4 and (b) FeVO4

The morphology of pure BiVO4 and FeVO4 was investigated by FESEM JEOL-7001F, in figure S1 the SEM images reveals the nanoparticles and rod like shaped morphology of BiVO4 and FeVO4, respectively.

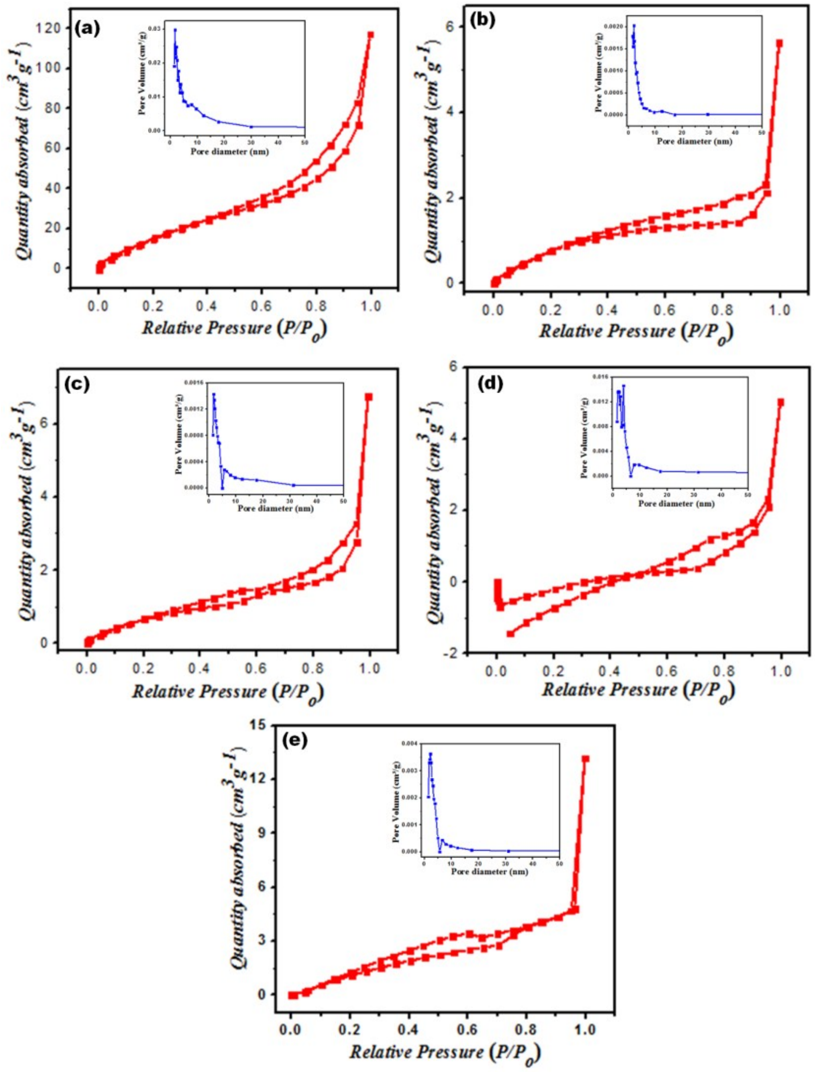

Figure. S2 Show the N_2 gas isotherms at 77.5 K of $BiVO_4/FeVO_4$ nanocomposite at a different mole ratio at **(a)** 1:5 **(b)** 1:2 **(c)** 1:1 **(d)** 5:1 and **(e)** 10:1 the BET surface area and BJH pore size in inset.

We find out existence of hysteresis loop in the all-inclusive $BiVO_4/FeVO_4$ nanocomposites, between the adsorption and desorption treat during N_2 isotherm; as in figure S1 all the materials belongs different adsorption type of curves to the H_2 and H_3 typical hysteresis; at concentration 1:5, 1:1 of $BiVO_4/FeVO_4$ corresponds to type III curve of H_3, at 1:2 and 5:1 concentration correspond to type II curve of H_3 and at concentration value 10:1 the type II curve of H_2. The H_2 and H_3 type is related to the parallel wall, slit shapes capillaries, particles aggregate and narrow bottle shape either both ends open or closed give lamellar centre structure and slit form pores[1 2]. The precipitous reduction in desorption isotherm of all $BiVO_4/FeVO_4$ flows from to cavitation and percolation.

Crystal Violet ($C_{25}H_{30}ClN_3$) a synthetic dye represents the mixing of tetramethyl, pentamethyl, hexamethyl and pararosanilines and normally utilized in fabric manufactures for colour purposes, has a triarylmethane structure as shown in figure S_2. It is toxic in nature as it gives toxic output as carbon monoxide, carbon dioxide, nitrogen oxides and hydrogen chlorides stimulate harmful effects on human, agrarian and on aquatic life [3 4].

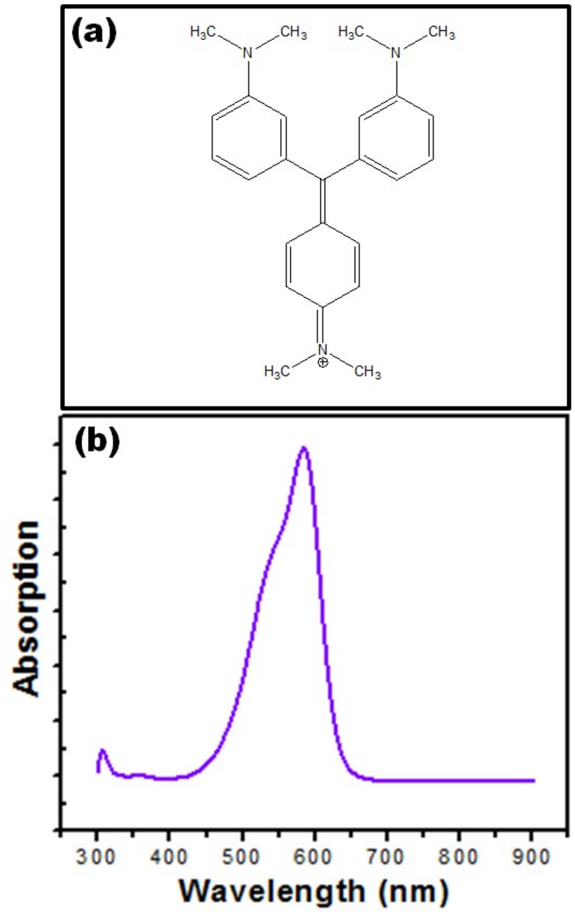

Figure S3 (a) Structure **(b)** adsorption spectrum of Crystal violet (CV) used as sample dye

The different mole ratio of $BiVO_4/FeVO_4$ heterojunction nanophotocatalyst affects the photocatalytic activity due to surface charge, particle size of nanostructures, it influence the absorption and/or desorption rate of dye which causes of the effect the photocatalytic response of the purposed materials[5]. Figure S3 (a-f) shows the spectra recorded for different mole ratios values of $BiVO_4/FeVO_4$ 1:5, 1:2, 1:1, 2:1, 5:1, 10:1 during the degradation of crystal violet dye solution at neutral pH value. It shows that at higher concentration of $FeVO_{4,}$ the degrading is higher in the beginning than decreases. When the $BiVO_4$ ratio is increased again the photocatalytic activity decreased. The highest degradation efficiency 99.1% at mole ratio 2:1 in 60 mints was recorded. It may be due to optimum ratio of $BiVO_4$ and $FeVO_4$ for the $BiVO_4/FeVO_4$ heterojunction nanophotocatalyst formation or may be due to morphology effect.

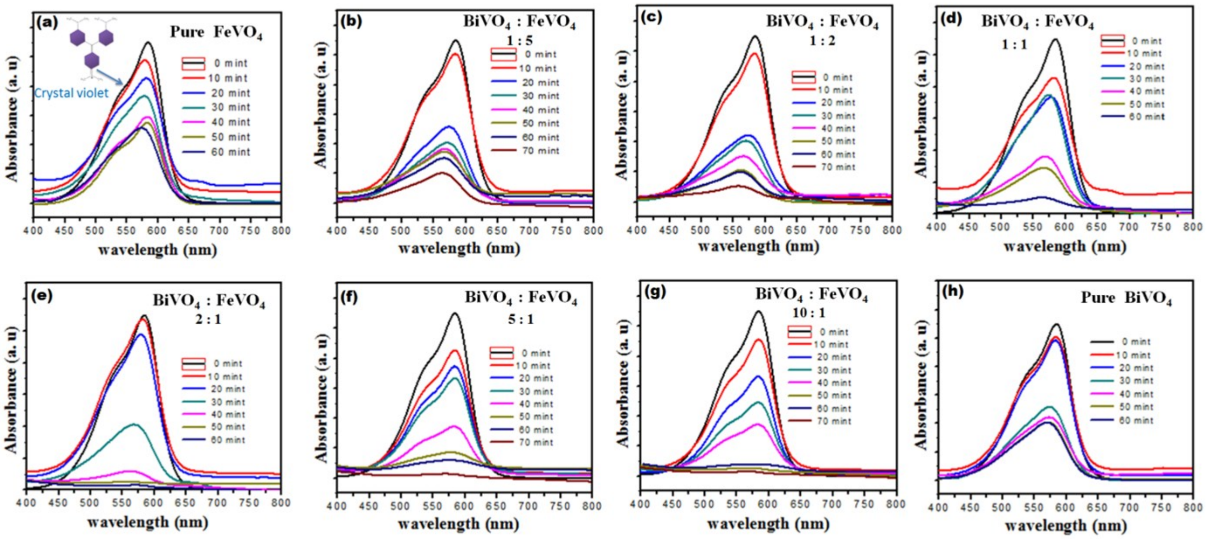

Figure S4 The photocatalytic degradation of Crystal violet dye by $FeVO_4$, $BiVO_4$ and $BiVO_4/FeVO_4$ hetro junction nanophotocatalyst at **(a)** Pure $FeVO_4$, **(b)** 1:5, **(c)** 1:2, **(d)** 1:1, **(e)** 2:1, **(f)** 5:1, **(g)** 10:1 mole ratio and **(h)** Pure $BiVO_4$.

From the above result and analysis it is observed that at 1:5 the degradation is 85 % in 70 mint, at 1:2 , 82 %, at 1:1, 98 % , at 2:1, 99.1 % , at 5:1, 97 % and at 10:1 is 98.6 %; the mole ratio 2:1 is the optimum concentration for $BiVO_4/FeVO_4$ in this study.

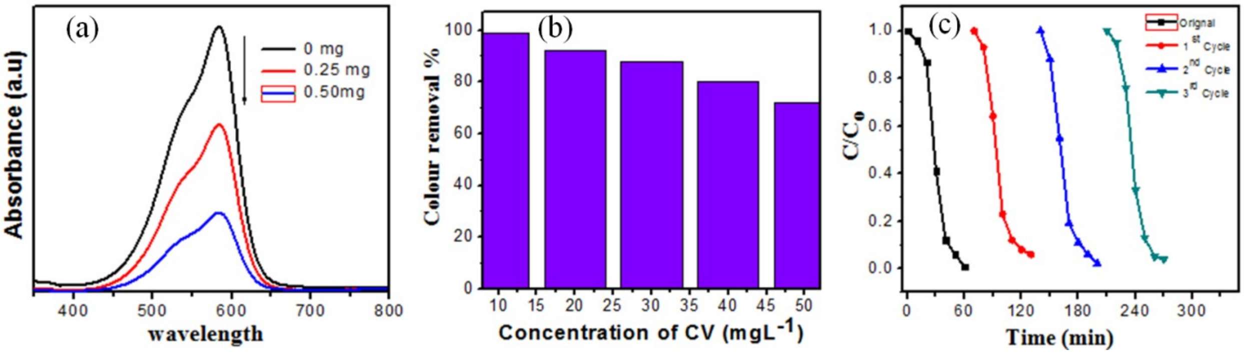

Figure. S5 (a) Absorption spectra of CV solution at different concentration of $BiVO_4/FeVO_4$ in 10mL CV solution, for 1h respectively. (b) Effect of the initial dye concentration on photocatalytic degradation of CV (c) Stability curves of the $BiVO_4/FeVO_4$ photocatalyst for CV dye under visible light

References:

1. D. V. Bavykin, V. N. Parmon, A. A. Lapkin and F. C. Walsh, *Journal of Materials Chemistry*, 2004, **14**, 3370-3377.
2. S. B. Khan, M. Hou, S. Shuang and Z. Zhang, *Applied Surface Science*, 2017, **400**, 184-193.
3. M. F. H. Al-Kadhemy, W. H. Abaas and I. Fakher, *Caspian Journal of Applied Sciences Research*, 2013, **2**.
4. S. Mani and R. N. Bharagava, in *Reviews of Environmental Contamination and Toxicology*, Springer, 2016, ***237,*** 71-104.
5. L. S. Zhang, T. F. Long, C. Q. Yan, X. C. He, M. L. Cheng and S. Zhong, *Advanced Materials Research*, 2013, **662**, 372-378.
